# Mechanochemical effects underlying the mechanically activated catalytic hydrogenation of carbon monoxide

**DOI:** 10.1038/s41598-023-28972-8

**Published:** 2023-02-11

**Authors:** Maria Carta, Anna Laura Sanna, Andrea Porcheddu, Sebastiano Garroni, Francesco Delogu

**Affiliations:** 1 Dipartimento di Ingegneria Meccanica, Chimica, e dei Materiali, CSGI Cagliari Research Unit, Università degli Studi di Cagliari, via Marengo 2, 09123 Cagliari, Italy; 2 Dipartimento di Scienze Chimiche e Geologiche, Università degli Studi di Cagliari, Cittadella Universitaria, SS 554 bivio per Sestu, 09042 Monserrato, CA Italy; 3 Dipartimento di Chimica e Farmacia, Università degli Studi di Sassari, Via Vienna 2, 07100 Sassari, Italy

**Keywords:** Heterogeneous catalysis, Design, synthesis and processing

## Abstract

In this work, we highlight and measure the intensity of mechanochemical effects at work in the hydrogenation of carbon monoxide by comparing the activity of a supported Co–Fe catalyst subjected, respectively, to ball milling and simple powder agitation. Paying due regard to the discontinuous nature of ball milling, we show that mechanochemical hydrogenation proceeds at significantly higher rate and disclose its connection with individual impacts. Experimental evidence suggests that the enhanced catalytic activity we observe can be ascribed to local processes affecting the amount of powder that gets involved in individual impacts.

## Introduction

Easy to operate and apparently simple, mechanical processing by ball milling (BM) is a versatile method to comminute, blend and react granular solids^1,2^. The powder particles trapped between two impacting milling tools are subjected to dynamic compaction at relatively high strain rates, experiencing heterogeneous force chains that spread across their contact points. As local mechanical stresses exceed the yield stress, particles deform^1,2^. Under the effects of an intense dislocation activity, new surfaces and interfaces are generated, while chemical species can undergo forced mixing and chemical combination^1–3^. This form of chemistry goes by the name of mechanochemistry^4^.

During the last 50 years, mechanochemical methods have enabled the fabrication of oxide-dispersion-strengthened superalloys^5^, amorphous metals^5–7^, and nanocrystalline materials^7–10^. Similarly, they have been used to ignite self-propagating high-temperature reactions^11^, facilitate gas absorption and desorption processes^12,13^, degrade pollutants^14,15^, improve mineral leachability^16,17^, and enhance biomass conversion^18,19^. More recently, mechanical processing paved the way to effective solvent-less synthetic routes to fine chemicals and pharmaceuticals^20–23^. This earned mechanochemistry the inclusion among the top ten chemical innovations likely to make chemical productions more sustainable^24^.

Due to its successful application to the preparation of new materials, and the activation and intensification of chemical processes, BM is generally regarded as an advantageous alternative to more conventional methods^25,26^. Nevertheless, comparison is made mainly on phenomenological basis. Typically, a given chemical reaction is shown to reach higher yields, or even complete conversion, under BM conditions, whereas its classical counterpart displays lower yields, or does not occur at all. While this suggests that BM can be sometimes superior, the quantitative assessment of its capabilities is substantially lacking.

Actually, it is almost 50 years that mechanochemistry defies scientists’ attempts to reveal its most intimate mechanisms. Before the clear evidence that mechanical forces and thermodynamic driving forces combine to give rise to a novel far-from-equilibrium chemistry, alternative chemical transformation paths and unexpected reaction products have been generically explained invoking elusive mechanochemical effects. While this claim finds justification in the Onsager reciprocal relations for irreversible processes, mechanochemical effects have remained in the background of the chemistry activated by mechanical forces, elusive and yet perceivable.

In this work, we precisely address this issue. Specifically, we evaluate the intensity of the mechanochemical effects, so frequently and commonly invoked to explain experimental evidence. To this aim, we have investigated how BM affects the catalytic hydrogenation of carbon monoxide (CO).

Three main features justify our choice. First, conventional CO hydrogenation involves a solid catalyst and gaseous species^27–29^, and its mechanochemical counterpart can be carried out under the same nominal pressure and temperature conditions, which is a fundamental requisite for a meaningful comparison. Second, the reaction between CO and hydrogen (H_2_) has been investigated since the 1920s because of its relevance for the Fischer–Tropsch synthesis, and catalysts with well-characterized activity are available^30–33^. Third, we studied the mechanochemical CO hydrogenation in the past^34,35^, which provides us the necessary background to take on the challenge of quantifying mechanochemical effects.

Taking advantage of previous studies, we have conducted experiments using a bi-metallic cobalt-iron (Co–Fe) catalyst supported on titanium dioxide (TiO_2_)^36–38^. We planned the experiments with the specific objective of unravelling the relationship between chemical conversion and BM conditions. To this aim, experiments were carried out using, separately, catalyst powders suitably activated under H_2_ and pristine catalysts never exposed to H_2_. Specifically, we evaluate the number of CO moles converted per impact during BM in the presence of a single milling ball and suitably compare it with the outcome of more conventional catalytic runs where catalyst powders are simply agitated inside the milling reactor with no milling ball. We show that, once BM dynamics is suitably considered, the mechanochemical process is orders of magnitude more efficient.

## Results

**Figure 1:**
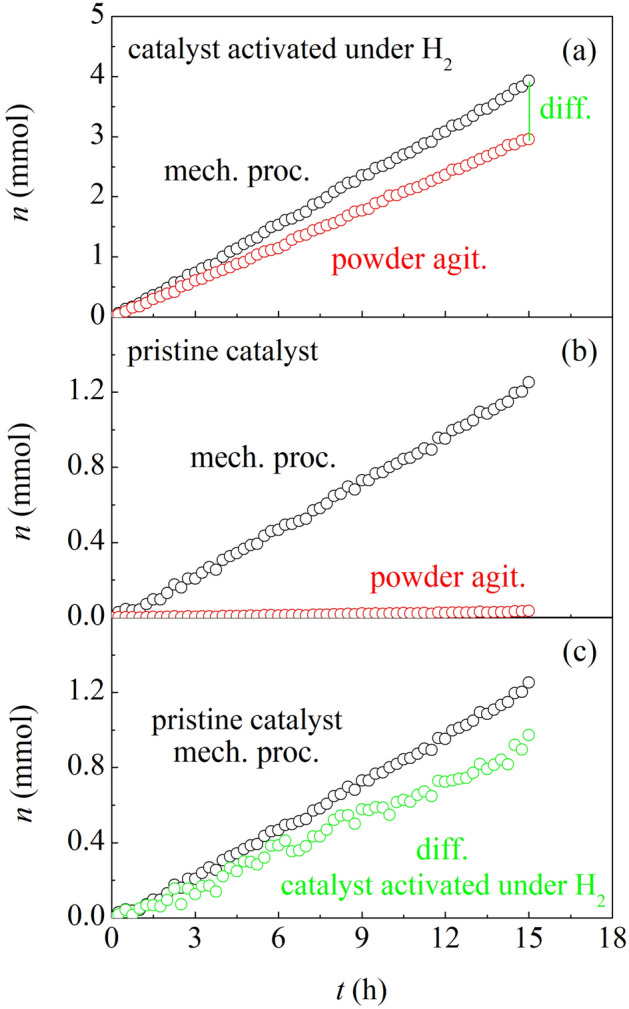
The number of CO moles reacted, $n$, as a function of time, $t$, under BM (black) and PA (red) conditions. Data refer to CO reacted over (**a**) catalyst powders activated under H_2_, and (**b**) pristine catalyst powders. (**c**) The number of CO moles, $n$, reacted over pristine catalyst powders subjected to BM (black) is compared with the difference in the number of CO moles, $\Delta n$, that react under BM and PA conditions over catalyst powders activated under H_2_ (green).

The present work revolves around the experimental evidence summarized in Fig. 1. The two datasets shown in Fig. 1a refer to experiments carried out using catalyst powders activated under H_2_ and exposed to a CO-H_2_ atmosphere (see Supporting Information SI.1 for details). Data simply consist of the number of CO moles reacted over the catalyst powders, $n$, as a function of time, $t$. They were obtained under BM and powder agitation (PA) conditions at room temperature. In both cases, $n$ varies linearly with $t$. The slopes of the linear plots indicate that the highest reaction rate goes with the catalytic process involving BM, where catalyst powders experience impacts (see Supporting Information SI.1 for details). In particular, the reaction rate over activated catalyst powders undergoing BM, ${r}_{act,BM}$, is equal to about 0.283 mmol h^−1^, whereas the reaction rate over activated catalyst powders undergoing PA, ${r}_{act,PA}$, is around 0.198 mmol h^−1^.

The data obtained from experiments carried out with pristine catalyst powders never activated under H_2_ are plotted in Fig. 1b (see Supporting Information SI.1 for details). Unlike the previous case, PA does not result in chemical reaction. In contrast, BM induces a linear $n$ increase at a rate ${r}_{prist,BM}$ of about 0.083 mmol h^−1^.

It appears that impacts enhance the activity of both activated and pristine catalyst powders. In this regard, the difference between the number of CO moles reacted over the catalyst powders activated under H_2_ and, then, subjected to BM and PA conditions, $\Delta n$, provides further insight. Indeed, data plotted in Fig. 1c show that $\Delta n$ increases linearly at a rate ${r}_{act,BM-PA}$ of about 0.081 mmol h^−1^. The ${r}_{act,BM-PA}$ and ${r}_{prist,BM}$ values are so similar that it is reasonable to infer that impacts have approximately the same effect on both activated and pristine catalyst powders.

**Figure 2:**
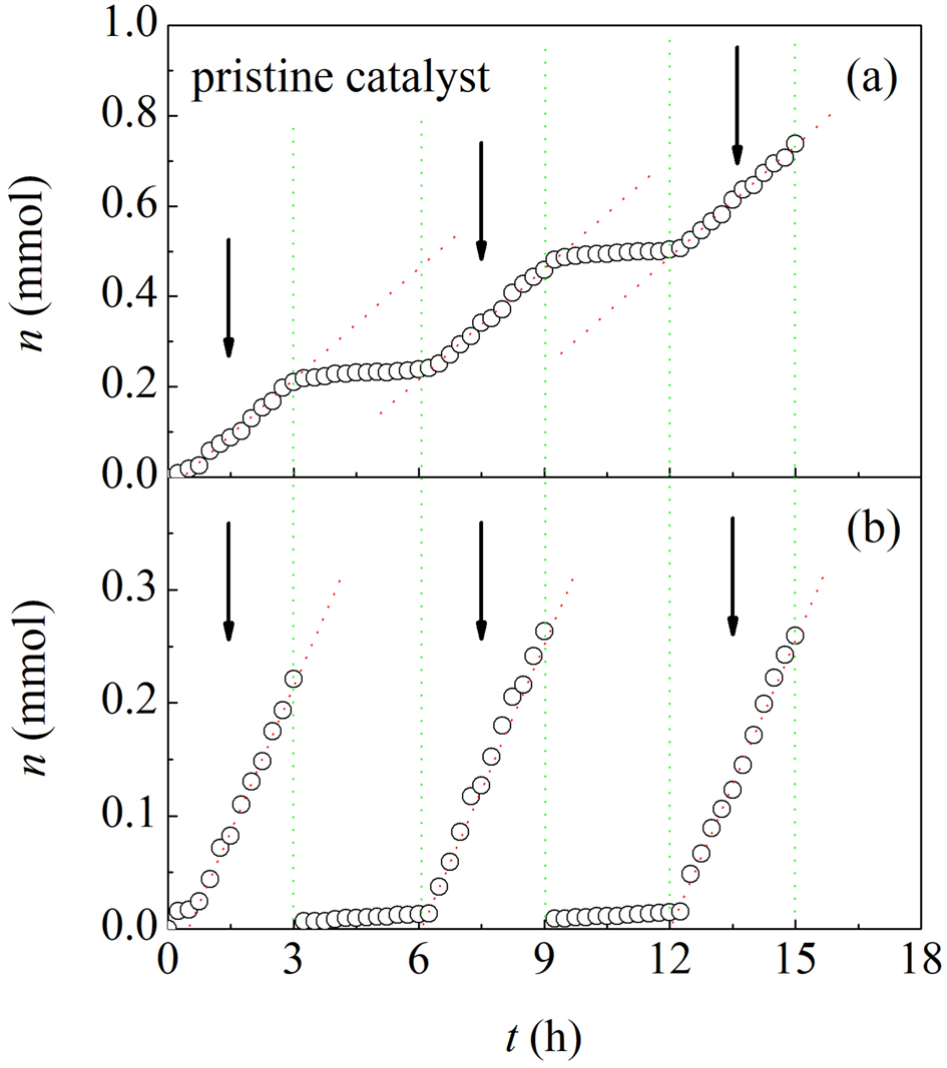
The number of CO moles reacted under BM conditions over pristine catalyst powders, $n$, as a function of time, $t$. Data refer to experiments in which (**a**) BM was interrupted and the vial never opened, and (**b**) BM was interrupted and the vial opened. Arrows indicate the time intervals during which the pristine catalyst powders were subjected to BM. Best-fitted lines (dotted, red) are shown.

In the light of such evidence, we carried out new experiments to further investigate the effect of impacts on pristine catalyst powder. To this aim, we have subjected pristine catalyst powders to BM for a time interval $\Delta t$, interrupted BM for the same time interval, and, then, re-started BM. We have repeated the cycle several times, using different time intervals $\Delta t$ (see Supporting Information SI.2 for details). Typical results are shown in Fig. 2a, where the number of CO moles reacted, $n$, is plotted as a function of time, $t$. It can be seen that $n$ increases linearly when catalyst powders undergo BM. Conversely, no chemical conversion is observed when BM is interrupted. Number of interruptions and $\Delta t$ length do not affect the reaction rate ${r}_{prist,BM}$, which remains equal to about 0.082 mmol h^−1^ in remarkable agreement with the ${r}_{prist,BM}$ values measured in previous experiments (see Supporting Information SI.2 for details).

In other experiments, we subjected pristine catalyst powders to BM for a given time interval $\Delta t$. Then, we interrupted BM, opened the reactor under inert atmosphere, and sealed it again, subsequently refilling it with the CO-H_2_ mixture (see Supporting Information SI.2 for details). Finally, we re-started BM for the same time interval. Representative data are shown in Fig. 2b. Again, $n$ increases linearly at a rate ${r}_{prist,BM}$ of about 0.085 mmol h^−1^. We observe that the $\Delta t$ length does not affect ${r}_{prist,BM}$ (see Supporting Information SI.2 for details).

Experimental evidence obtained so far indicates that the catalytic reaction proceeds only when pristine catalyst powders undergo BM and it immediately stops when BM is interrupted. We conclude that the catalytic activity exhibited by pristine catalyst powders can be ascribed to impacts and that the overall CO hydrogenation reaction can be regarded as the sum of independent contributions from individual impacts. Under these circumstances, our attention has to shift necessarily from continuous to discrete, i.e. from time $t$ to the number of impacts, $m$.

**Figure 3:**
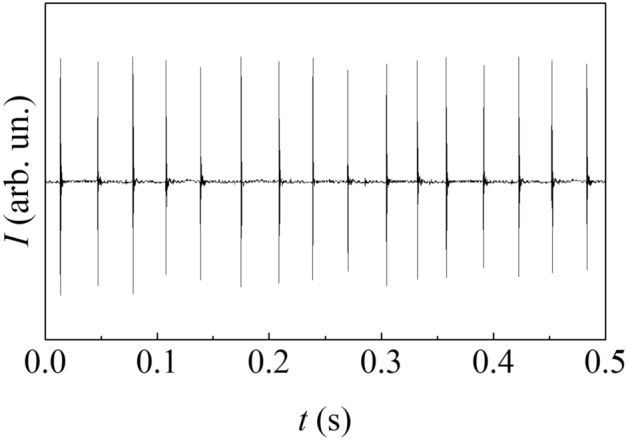
The intensity, $I$, of signals generated by the piezoelectric sensor as a function of time, $t$. Data refer to a BM experiment carried out swinging the reactor at 14.6 Hz.

The quantity that allows this transition is the impact frequency, $f$. Once $f$ is known, $m$ can be calculated by the product $f t$. Particularly in the presence of a single milling ball, $f$ can be easily measured equipping the reactor with piezoelectric sensors able to detect any impact between milling ball and reactor^39–41^ (see Supporting Information SI.3 for details). A representative sequence of piezoelectric signals is shown in Fig. 3.

An average impact frequency $f$ of about 29.3 Hz can be evaluated from the analysis of similar sequences. This value is approximately twice the reactor swing frequency, which corresponds to 14.6 Hz. Then, two impacts per reactor swing cycle take place^39–41^ (see Supporting Information SI.3 for details).

With this information, we can readily calculate the average number of CO moles reacted over pristine catalyst powders due to individual impacts, ${n}_{imp}$. This quantity amounts to about 7.89 × 10^–7^ mmol, and its evaluation represents the first step to properly compare the catalytic activity induced in pristine catalyst powders by impacts and the catalytic activity exhibited by activated catalyst powders under PA conditions.

To this aim, we note that the reaction of ${n}_{imp}$ CO moles induced by a single impact can be expected to require a characteristic time, $\tau$. The knowledge of $\tau$ would allow evaluating the reaction rate associated with individual impacts, ${r}_{imp}$, as the ratio between ${n}_{imp}$ and $\tau$. Unfortunately, measuring $\tau$ is out of reach. We can only roughly estimate its lower and upper bounds. The lower bound can be identified with the impact duration. Theoretical and experimental evidence for elastic impacts suggests that it ranges between 0.01 and 0.1 ms depending on the material of which impacting bodies are made^42–45^. The presence of a powder layer between them extends the impact duration, and the effect depends on the layer thickness^46^. A value of about 1 ms represents a reasonable choice in our case, although probably in excess. As for the upper bound, literature suggests that mechanical energy deposition into impacted powder can result in a chemical reactivity enhancement for time intervals as long as 20 ms^47^. Therefore, 50 ms seem to be a reasonable characteristic time, again in excess.

Based on such considerations, we assume that the characteristic reaction time $\tau$ ranges between 1 and 50 ms. Accordingly, the reaction rate associated with individual impacts, ${r}_{imp}$, ranges between 0.057 and 2.84 mmol h^−1^. Although ${r}_{imp}$ represents the mechanochemical reaction rate, it cannot be directly compared with the rate of the CO hydrogenation reaction over activated catalyst powders under PA conditions, ${r}_{act,PA}$. Indeed, while the whole catalyst powder participates in the catalytic process in the latter case, the reaction of ${n}_{imp}$ CO moles per impact can be ascribed only to the amount of catalyst powder involved in the impact. Therefore, we have to compare the specific reaction rates, which can be calculated by dividing the reaction rates by the suitable mass of catalyst powder.

Since the CO hydrogenation reaction over activated catalyst powders under PA conditions is carried out using 8 g of powder, the specific CO hydrogenation rate, ${r}_{act,PA,sp}$, is equal to about 2.45 × 10^–5^ mol g^−1^ h^−1^. In contrast, only about 0.63 mg of catalyst powder are involved in individual impacts (see Supporting Information SI.4 for details). Then, the specific reaction rate associated with individual impacts, ${r}_{imp,sp}$, approximately ranges between 0.090 and 4.508 mol g^−1^ h^−1^.

The difference in catalytic activity between activated catalyst powders subjected to PA and pristine catalyst powders subjected to impacts ranges between 3 and 6 orders of magnitude. Precisely this difference provides a measure of the mechanochemical effects stemming from the occurrence of impacts.

Concerning their nature, little can be said. BM does not induce any evident modification of the Co-Fe solid solution structure and microstructure. The same is true for the TiO_2_ support, which remains unaffected by prolonged BM (see Supporting Information SI.5 for details). Furthermore, control experiments with only TiO_2_ powders do not reveal any detectable chemical conversion of the reactant gaseous mixture (see Supporting Information SI.5 for details). Therefore, we conclude that the observed enhancement of catalytic activity induced by impacts can be explained by a corresponding enhancement of the catalyst surface reactivity.

To give at least an approximate idea of what this means, let us assume that CO hydrogenation under PA conditions exhibits Arrhenius-like behavior. Literature suggests that typical activation barriers are between 150 and 200 kJ mol^−1^^31,48–51^. An increase of 3–6 orders of magnitude in reaction rate can result from a reduction of the activation barrier, ${E}_{a}$, by less than 50 kJ mol^−1^, or an increase of temperature, $T$, up to 500 K, or a combination of these two factors (see Supporting Information SI.5 for details). Both the energy barrier reduction and the local temperature rise appear reasonable and compatible with the disordering and heating processes caused by impacts.

**Figure 4:**
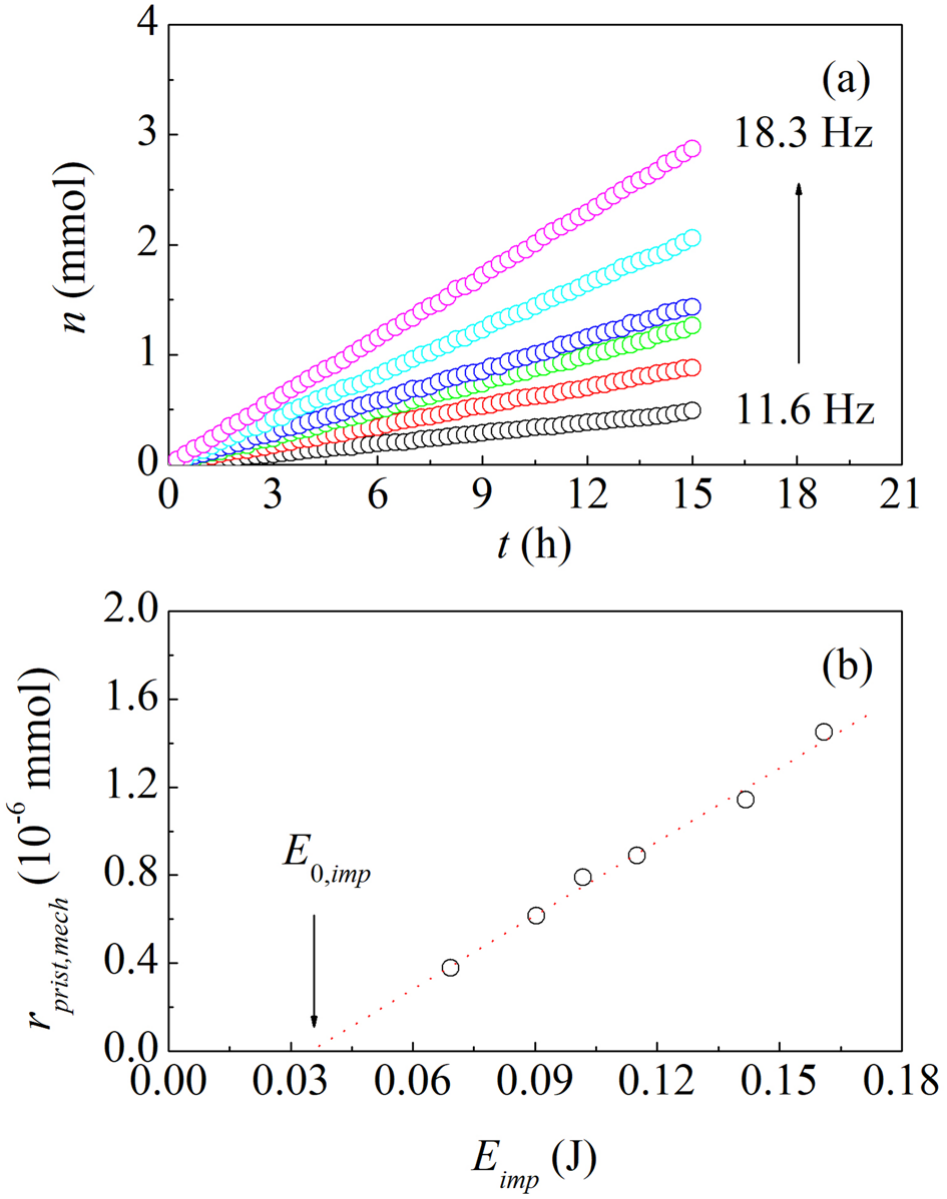
(**a**) The number of CO moles reacted, $n$, as a function of time, $t$, during ball milling in the presence of a single milling ball. Data refer to CO reacted over pristine catalyst powders never exposed to H_2_ for catalyst activation purposes. The different datasets were obtained using a milling frequency of 11.6 Hz (black), 13.3 Hz (red), 14.6 Hz (green), 15.0 Hz (blue), 16.6 Hz (cyan), and 18.3 Hz (magenta). (**b**) The number of CO moles reacted over pristine catalyst powders during individual impacts, ${n}_{prist,imp}$, as a function of the impact energy, ${E}_{imp}$. The best-fitted line is shown (dotted, red). The minimum impact energy required to activate the reaction, ${E}_{0,imp}$, is indicated.

We further notice that the catalytic activity of pristine catalyst powders exhibits an evident dependence on BM conditions. We changed the milling frequency in the range approximately between 11.6 and 18.3 Hz (see Supporting Information SI.3 for details). Accordingly, the impact energy, i.e. the energy transferred by the ball to the powders during each impact, ${E}_{imp}$, changed between 0.070 and 0.162 J. As shown in Fig. 4a, the impact energy definitely affects the CO hydrogenation rate. The linear change of the number of CO moles reacted, $n$, with the number of impacts, $m$, becomes increasingly steeper as ${E}_{imp}$ increases.

It can be readily seen from Fig. 4b that ${r}_{prist,BM}$ varies linearly with ${E}_{imp}$. The experimental points can be best-fitted by a line that crosses the abscissa axis at a finite, positive value of about 0.035 J, hereafter denoted as ${E}_{0,imp}$. Therefore, the best-fitted line is described by the equation

1 $$ r_{prist,BM} = \rho \left( {E_{imp} - E_{0,imp} } \right), $$

where $\rho$, equal to about 11.2 × 10^–6^ mmol J^−1^, can be interpreted as the number of CO moles reacted over pristine catalyst powders per unit impact energy. Equation (1) suggests that the CO hydrogenation over pristine catalyst powders requires a minimum impact energy ${E}_{0,imp}$. In fact, no reaction is observed when ${E}_{imp}$ is below such threshold. We relate the minimum impact energy to the occurrence of specific, not yet identified, mechanical deformation processes related to local contact mechanics on the microscopic scale.

Although the ultimate reasons behind our results require deeper and more specific investigations on the scale of individual impacts, we can definitely claim that mechanochemical effects are clearly discernible and measurable, determining for activated and pristine catalyst powders a catalytic activity enhancement of several orders of magnitude. The linearity of catalytic conversion during the mechanical processing of the catalyst, regardless it was previously activated or not using H_2_, is also worth noting. Both the mechanical processing by ball milling and the catalytic CO hydrogenation are, indeed non-linear processes, so that one can reasonably expect that their combination does not give rise to perfectly linear effects. However, the rationale for our observations has to be seeked, once again, in the processes activated, on the microscopic scale, by individual impacts. We are convinced that developing suitable methodologies to compare mechanically activated reactions with more conventional counterparts and the systematic quantification of mechanochemical effects can potentially open a new season of study in the field.

## Materials and methods

Catalyst powders were prepared using high-purity elemental Co and Fe powders, and anatase TiO_2_ powders. A nanocrystalline equimolar chemically-disordered solid solution was fabricated by mechanical alloying. The resulting Co_50_Fe_50_ powders were finely dispersed on anatase TiO_2_ powders by mechanical processing. In all cases, experiments were carried out under Ar atmosphere utilizing stainless-steel balls and a stainless-steel vial swung by a SPEX Mixer/Mill 8000. Final powder particle size was measured with a Malvern Zetasizer nano s90.

Catalytic runs were carried out using catalyst powders as prepared and after suitable activation. To this aim, pristine catalyst powders were exposed for 48 h to a gaseous H_2_ flow at the pressure of 1 MPa and the temperature of 450 K.

The formation of the nanocrystalline Co_50_Fe_50_ solid solution by mechanical alloying and its subsequent dispersion on anatase TiO_2_ powders by mechanical processing were monitored by wide-angle X-ray diffraction (XRD). XRD patterns were analyzed with the Rietveld method. Total specific surface area was measured by N_2_ physisorption according to the BET method. Adsorption curve reveal non-porous structure. The specific surface area of the catalytically active Co_50_Fe_50_ solid solution phase was measured by H_2_ and CO chemisorption. Scanning and transmission electron microscopy (SEM and TEM) were used to estimate the characteristic size of the catalytically active Co_50_Fe_50_ phase.

CO hydrogenation reactions were carried out over pristine catalyst powders and catalyst powders activated under H_2_. In both cases, 8 g of catalyst powders were used. The catalyst powders were placed inside a stainless-steel vial sealed by a cap equipped with gas-tight pressure valves for the inlet and outlet of gases. The initial Ar atmosphere was replaced by the desired 1:3 CO:H_2_ gaseous mixture via several purge-refill cycles. Gas pressure was set at 0.3 MPa. All the experiments were performed at room temperature.

Two types of experiments were performed. On the on hand, pristine catalyst powders or catalyst powders activated under H_2_ were simply agitated. To this aim, no milling ball was placed inside the vial swung by the SPEX Mixer/Mill 8000. On the other hand, pristine catalyst powders or catalyst powders activated under H_2_ were subjected to mechanical processing in the presence of a single 12-g stainless-steel ball. In this latter case, milling dynamics was monitored using a piezoelectric transducer suitably stuck to the stainless-steel vial bottom base.

Control experiments were performed using anatase TiO_2_ powders.

The gaseous mixture inside the vial was analyzed every 15 min by gas-chromatography (GC) using a Fisons 8000 gas-chromatograph equipped with a HWD detector. The gas was sampled automatically by a gas valve placed on the cap of the stainless-steel vial and injected into the GC column. Hydrocarbons formed by CO hydrogenation, mostly methane, were monitored in independent experiments with a Perkin Elmer 8600 gas-chromatograph equipped with a FID detector. Gaseous species were identified based on the comparison of characteristic retention times of commercial standards with experimental ones. Absolute and relative CO, H_2_ and hydrocarbon amounts were determined by peak-area evaluation.

## Supplementary Information

Supplementary Information.

## Data Availability

Datasets generated and analysed during the current study cannot be shared at this time as they also forms part of an ongoing study. However, they can be available totally or in part from the corresponding author on reasonable request.
