# Supplementary material for: Mechanochemical effects underlying the mechanically activated catalytic hydrogenation of carbon monoxide
Source: Sci Rep. 2023 Feb 11;13:2470. doi: 10.1038/s41598-023-28972-8 (PMC9922255; doi:10.1038/s41598-023-28972-8)
Supplement: Supplementary file 1 — Supplementary Information. [file 41598_2023_28972_MOESM1_ESM.pdf]

Mechanochemical effects underlying the mechanically activated catalytic hydrogenation of carbon monoxide

Maria Carta[1], Anna Laura Sanna[1,2], Andrea Porcheddu[2], Sebastiano Garroni[3], Francesco Delogu[1,*]

[1] Dipartimento di Ingegneria Meccanica, Chimica, e dei Materiali, Università degli Studi di Cagliari, CSGI research unit, via Marengo 2, 09123 Cagliari, Italy

[2] Dipartimento di Scienze Chimiche e Geologiche, Università degli Studi di Cagliari, Cittadella Universitaria, SS 554 bivio per Sestu, 09042, Monserrato (CA), Italy

[3] Dipartimento di Chimica e Farmacia, Università degli Studi di Sassari, via Vienna 2, 07100 Sassari, Italy

Supporting Information

SI.1. Experimental methods

Catalyst powders were prepared using high-purity Co (< 150 μm, > 99.9%), Fe (< 60 μm, > 99.0%), and anatase TiO_2 (-325 mesh, > 99.0%) powders purchased from Sigma-Aldrich. Powders were stored and handled in a glove box under Ar inert atmosphere with oxygen and humidity contents below 1 ppm.

SI.1.1. Mechanical alloying of Co and Fe elemental powders

An equimolar mixture of Co and Fe powders was prepared manually and a stainless-steel cylindrical vial of about 65 ml in volume was loaded with a 10-g aliquot of powder together with two 12-g stainless-steel balls. Once sealed under Ar atmosphere, the reactor was clamped on the mechanical arm of a SPEX Mixer/Mill 8000 and swung at a milling frequency of about 14.6 Hz.

After 50 h, the mechanical alloying of elemental powders was complete and a nanocrystalline $Co_{50}Fe_{50}$ solid solution was obtained.

A Malvern Zetasizer nano s90 was used to measure the particle size of final powders. These were affected by significant aggregation, only partly overcome by ultrasound sonication. The mechanical processing determined a significant particle comminution. The average particle size was around 300 nm, but finest particles were about 50 nm in size.

The formation of the nanocrystalline $Co_{50}Fe_{50}$ solid solution by mechanical alloying was monitored by wide-angle X-ray diffraction (XRD). Powders were compacted into thin disk specimens and analyzed using a Rigaku SmartLab diffractometer equipped with a PhotonMax high-flux 9 kW rotating anode X-ray source. The XRD pattern of the final nanocrystalline solid solution is shown in Fig. SI.1.1.1.

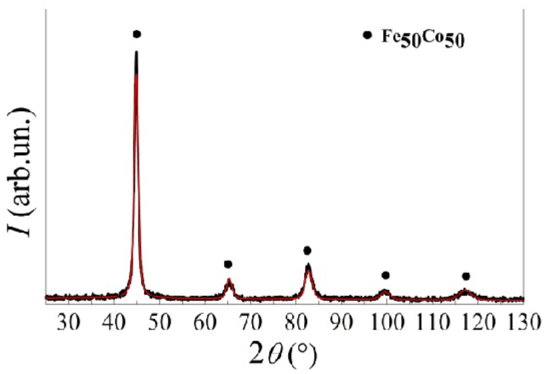

Fig. SI.1.1.1. XRD pattern of the nanocrystalline solid solution obtained after 50 h of mechanical alloying (black dots). The best-fitted integral profile obtained by using the Rietveld refinement is also shown (red line).

The experimental XRD patterns were quantitatively analyzed using the Rietveld method. It appears that mechanical alloying induces the progressive mutual dissolution of the elemental species and the formation of a nanocrystalline solid solution with average grain size of about 30 nm. Peak positions also indicates that the solid solution exhibits equiatomic composition on the

average. The gradual change of the unit cell parameters of both elements and solid solution suggests the formation of a chemically disordered phase.

Scanning electron microscopy (SEM) was carried out on suitably prepared powder samples using a Zeiss EVO LS15 microscope. The particle size was estimated from 10 SEM micrographs using a common image analysis software. On the average, particles were around 200 nm, but finer particles of about 20 nm were observed.

SI.1.2. Preparation of $(Co_{50}Fe_{50})_{0.2}(TiO_2)_{99.8}$ catalyst powders

A $(Co_{50}Fe_{50})_{0.2}(TiO_2)_{99.8}$ powder mixture was prepared mixing suitable amounts of $Co_{50}Fe_{50}$ and anatase TiO_2 powders. A stainless-steel cylindrical vial was loaded with a 10-g aliquot of such powder mixture and two stainless-steel 12-g balls. The vial was sealed under Ar atmosphere, clamped on the mechanical arm of a SPEX Mixer/Mill 8000 and swung at about 14.6 Hz for 80 h. In this way, the metallic phase was finely dispersed on the TiO_2 support.

The choice of working with a catalyst containing much less metallic phase, compared with the support, than usual is related to two main reasons. On the one hand, reducing the amount of metallic phase prevents aggregation and cold welding processes that typically result in the unsatisfactory dispersion of the metallic phase on the support due to the large size of metallic particles. On the other, we had to use a relatively large amount of powder inside the reactor to enable the fine control of the milling dynamics and, at the same time, we had to keep the CO hydrogenation rate relatively low to obtain accurate measurement of the conversion degree. Since the CO hydrogenation is carried out under batch conditions within a relatively small volume, the only way to control the reaction rate was to reduce the amount of the catalytically active metallic phase.

The particle size of final powders, d, was measured using a Malvern Zetasizer nano s90. Aggregation processes were still at work. However, this time ultrasound sonication was able to break most of aggregates. The obtained size distribution, $p(d)$, is shown in Fig. SI.1.2.1a. The distribution is narrow, with average size around 70 nm and finest particles around 50 nm.

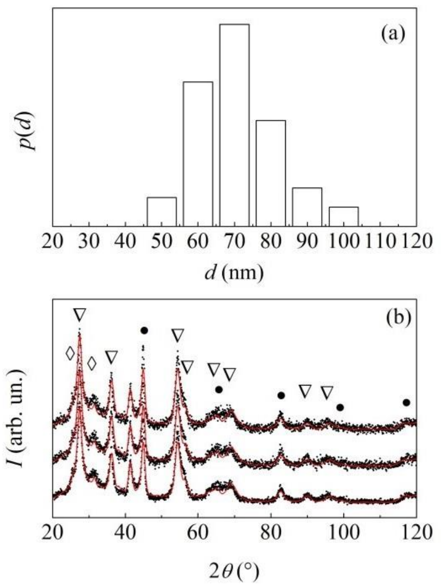

Fig. SI.1.2.1. (a) The particle size distribution of catalyst powders, $p(d)$, after 80-h mechanically activated dispersion of the metallic phase on the TiO_2 support. (b) XRD patterns of catalyst powders after (from bottom to top) 80-h, 120-h and 160-h mechanically activated dispersion of the metallic phase on the TiO_2 support. The intensity of scattered radiation, I, is plotted as a function of the scattering angle, 2θ. Peaks belong to Fe-Co solid solution (●), TiO_2 rutile (□) and TiO_2 brookite (◊).

A suitable amount of the final $(Co_{50}Fe_{50})_{0.2}(TiO_2)_{99.8}$ catalyst powder was compacted into thin disk specimens and placed inside a special sample holder that allows carrying out XRD under Ar atmosphere. A Rigaku SmartLab diffractometer equipped with a PhotonMax high-flux 9 kW rotating anode X-ray source was used. The XRD pattern of final powders is shown in Fig. SI.1.2.1b. Three different nanocrystalline phases are present, namely the $Co_{50}Fe_{50}$ solid solution with grain size around 20 nm, and TiO_2 rutile and TiO_2 brookite with grain size of about 50 nm and 40 nm respectively. The TiO_2 anatase has been completely transformed into the two TiO_2 polymorphs stable at higher temperatures.

The XRD patterns collected after 120 h and 160 h of ball milling are also shown in Fig. SI.1.2.1b. They are identical to the XRD patterns collected after 80 h. Therefore, the

$(Co_{50}Fe_{50})_{0.2}(TiO_2)_{99.8}$ powder mixture exhibits stable structure and microstructure, and the mechanical processing is unable to induce further structural and microstructural changes.

Suitably prepared $(Co_{50}Fe_{50})_{0.2}(TiO_2)_{99.8}$ powder samples were examined by SEM. SEM micrographs were collected using a Zeiss EVO LS15 microscope. A typical SEM micrograph is shown in Fig. SI.1.2.2a. The particle size distribution was estimated from 10 SEM micrographs using a common image analysis software. The results are shown in Fig. SI.1.2.2b. While the average particle size is around 70 nm, the finest particles have size around 40 nm.

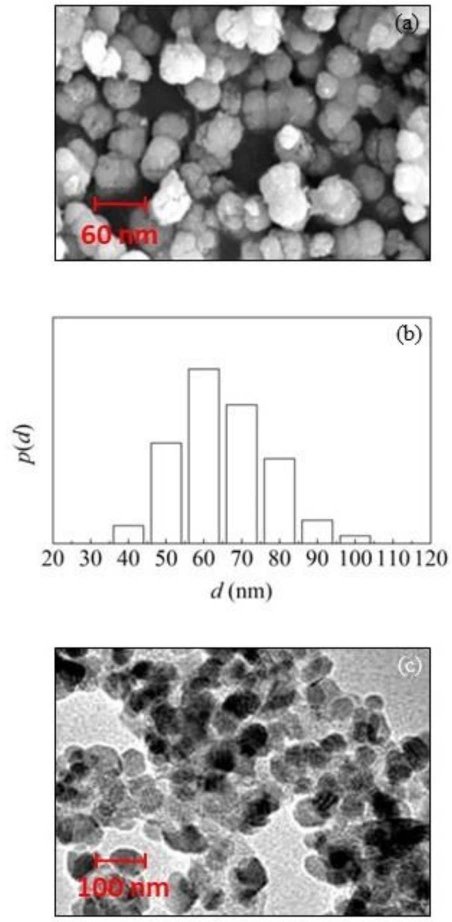

Fig. SI.1.2.2. (a) SEM image, (b) particle size distribution, $p(d)$, and (c) TEM image of catalyst powders after 80-h mechanically activated dispersion of the metallic phase on the TiO_2 support. Darker areas correspond to the Co-Fe metallic phase.

Transmission electron microscopy (TEM) was utilized to observe the metallic particles dispersed on the support. TEM micrographs were collected using a FEI Tecnai G12 microscope on a few selected $(Co_{50}Fe_{50})_{0.2}(TiO_2)_{99.8}$ powder samples. The representative TEM micrograph reported in Fig. SI.1.2.2c shows that the catalyst particles consist of $Co_{50}Fe_{50}$ particles with size between 20 nm and 30 nm dispersed over the TiO_2 on support particles with size around 60 nm.

The specific surface area of the catalyst powders was measured by low-temperature N_2 adsorption according to the Brunauer-Emmett-Teller (BET) method. A BET Fisons Instrument apparatus was used. A suitable amount of powder was placed inside the sample cell and outgassed applying, first, vacuum, and, then, purging the sample in a flowing stream of Ar gas. The Ar atmosphere was subsequently replaced with N_2 and the sample subjected to adsorption measurements according to the classical volumetric method. The $(Co_{50}Fe_{50})_{0.2}(TiO_2)_{99.8}$ powders exhibited a specific surface area of about 56 m^2 g^{-1}. The shape of isothermal adsorption curves is characteristic of non-porous powders.

The specific surface area of the catalytically active $Co_{50}Fe_{50}$ phase was measured by H_2 and CO chemisorption using a 3P Instruments AMI-300 analyzer. A suitable amount of powder was introduced in the sample cell. Once outgassing was complete, vacuum conditions were imposed. Then, the desired gas was dosed onto the sample. A first adsorption isotherm was evaluated, measuring the joint contribution of physisorption and chemisorption. Afterwards, the sample cell was evacuated to allow the slow desorption of the physisorbed gas from the particle surface. The comparison between first and second isotherms allowed to evaluate the contribution of pure chemisorption processes.

SI.1.3. Catalytic runs

Experiments were carried out using two sets of catalyst powders, namely pristine catalyst powders used as prepared and catalyst powders subjected to surface activation under H_2 atmosphere. In this latter case, the powders were placed in a large alumina vessel to form a thin layer and the vessel introduced in a furnace. The furnace temperature was raised up to 450 K and the powders exposed to a H_2 flux at a pressure of 1 MPa for 48 h.

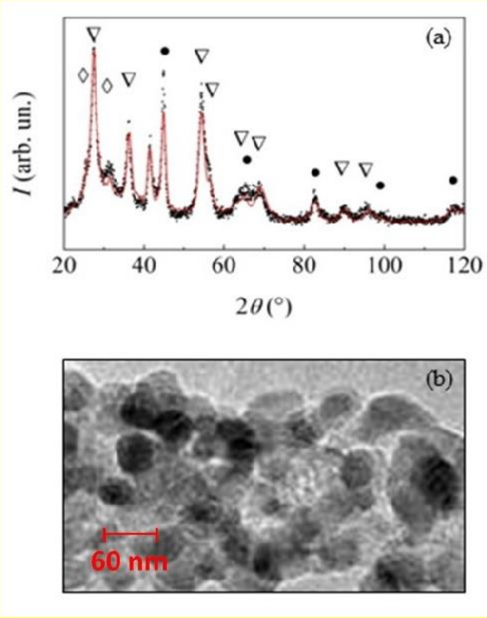

Fig. SI.1.3.1. (a) XRD pattern of catalyst powders after activation under H_2. The intensity of scattered radiation, I, is plotted as a function of the scattering angle, 2θ. Peaks belong to Fe-Co solid solution (●), TiO_2 rutile (□) and TiO_2 brookite (◊). (b) TEM image of catalyst powders after activation under H_2.

The activation conditions were suitably chosen not to induce structural and microstructural changes. This is evident from the XRD pattern shown in Fig. SI.1.3.1a, which is substantially identical to the ones reported in Fig. SI.1.1.1b. TEM micrographs such as the one shown in Fig. SI.1.3.1b confirm that activated catalyst powders are indistinguishable from pristine ones.

SEM observation confirms the compositional homogeneity of catalyst powders. We used, in particular, energy-dispersive X-ray (EDX) spectroscopy to obtain a satisfactory mapping of elemental species on the length scale of 10 nm. A typical SEM image and the spatial distribution of elements through chemical mapping are shown in Fig. SI.1.3.2. The chemical mapping of elemental species by EDX indicates that the metallic phase has equiatomic composition.

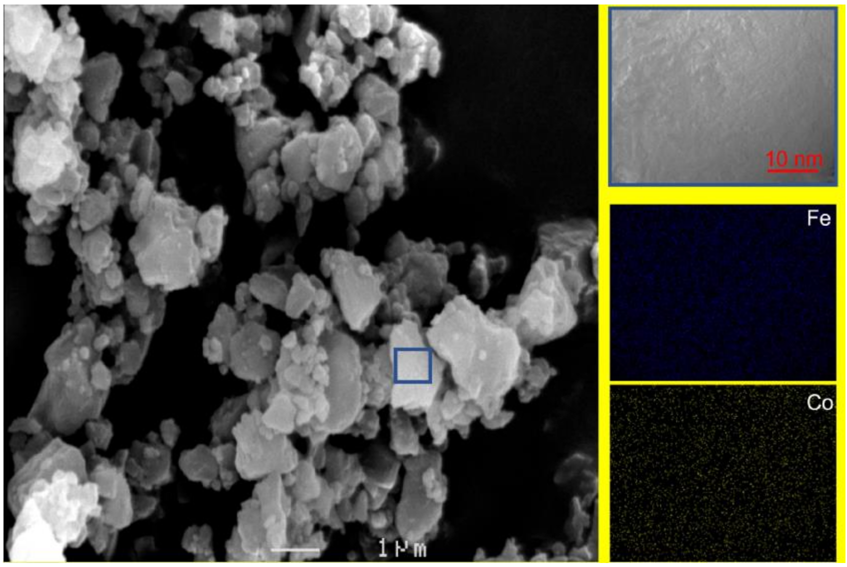

Fig. SI.1.3.2. (left) SEM image of catalyst powders after activation under H2. (right) Detail of the SEM image on a length scale of 10 nm (top), and the associated EDX chemical mapping for Fe (middle) and Co (bottom).

CO hydrogenation reactions were carried out using 8 g of pristine or activated catalyst powders. The powder was placed inside a stainless-steel vial under Ar atmosphere. The reactor was sealed using a cap equipped with gas-tight pressure valves for the inlet and outlet of gases. Through several purge-refill cycles, the initial Ar atmosphere was replaced by a $CO:H_2$ gaseous mixture with composition 1:3. The final gas pressure was set at 0.3 MPa.

Catalytic runs were carried out without and with a milling ball. In the former case, pristine or activated catalyst powders were simply subjected to agitation within the reactor swung by the SPEX Mixer/Mill 8000. In the latter case, pristine or activated catalyst powders were subjected to mechanical processing in the presence of a single 12-g stainless-steel ball.

The atmosphere inside the reactor was analyzed every 15 min using a Fisons 8000 gas-chromatograph equipped with a HWD detector. The gas was sampled automatically by a gas valve placed on the cap of the stainless-steel vial and injected into the GC column. Hydrocarbons formed by CO hydrogenation were monitored in independent experiments with a Perkin Elmer 8600 gas-chromatograph equipped with a FID detector. Gaseous species were identified

comparing the characteristic retention times of experimental gas-chromatograms with those of commercial standards. Absolute and relative CO, H_2 and hydrocarbon amounts were determined by peak-area evaluation.

SI.2. Additional and control milling experiments

Catalytic runs have been carried out under different processing conditions with the aim of clarifying the role of impacts and their effects on the activity of catalyst powders. Along the same line, experiments have been also performed in the presence of TiO_2 powders alone to rule out any contribution to CO hydrogenation due to the support phase.

SI.2.1. Additional milling experiments

CO hydrogenation reactions were performed using 8 g of pristine catalyst powders. The powder was placed inside a stainless-steel vial under Ar atmosphere together with a 12-g stainless-steel ball. The reactor was sealed using a cap equipped with gas-tight pressure valves for the inlet and outlet of gases. Through several purge-refill cycles, the initial Ar atmosphere was replaced by a CO:H_2 gaseous mixture with composition 1:3. via. The final gas pressure wasset at 0.3 MPa. Once clamped to the mechanical arm, the reactor was swung by the SPEX Mixer/Mill 8000.

In a first set of experiments, pristine catalyst powders were subjected to BM for a time interval Δt, interrupted BM for the same time interval, and, then, re-started BM. The cycle was repeated several times. Different time intervals Δt were used to investigate if the length of the interruption period affects the catalyst powder activity. In all the different cases investigated, the number of CO moles reacted, n, increases linearly with time when catalyst powders undergo BM. Conversely, no chemical conversion is observed when BM is interrupted. As shown in Fig. SI.2.1a, the Δt length does not affect the reaction rate $r_{\text{prist,BM}}$. Indeed, the $r_{\text{prist,BM}}$ values keep approximately constant around 0.082 mmol h^{-1}.

In a second set of experiments, pristine catalyst powders were subjected to BM for a given time interval Δt. Once the BM was interrupted, the reactor was opened under inert Ar atmosphere, and sealed again after a few minutes. Then, the reactor was refilled with a CO:H_2 gaseous mixture with composition 1:3, and the final gas pressure was set at 0.3 MPa. Finally, BM was re-started for the same time interval. In all the different cases, the number of CO moles reacted, n, increases linearly with time during the BM stage, while no conversion is observed

during the BM interruption periods. Data shown in Fig. SI.2.1b indicate that the Δt length does not affect the reaction rate $r_{\text{prist,BM}}$. Indeed, the $r_{\text{prist,BM}}$ values keep approximately constant around 0.084 mmol h^{-1}.

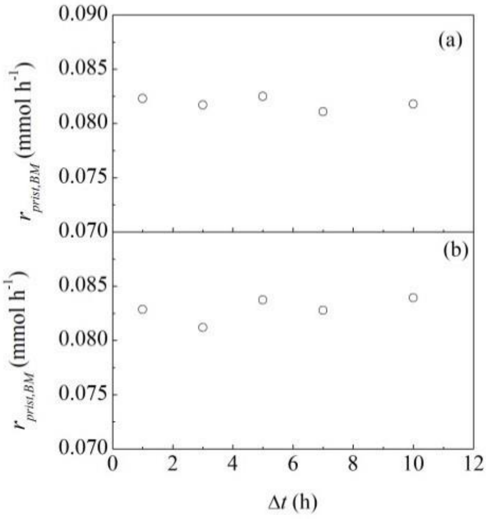

Fig. SI.2.1. (a) The CO hydrogenation rate over pristine catalyst powders, $r_{\text{prist,BM}}$, as a function of the processing time interval, Δt. The reactor was not opened. (b) The CO hydrogenation rate over pristine catalyst powders, $r_{\text{prist,BM}}$, as a function of the processing time interval, Δt. The reactor was opened under inert Ar.

SI.2.2. Control milling experiments

Anatase TiO_2 powder was subjected to BM under the same processing conditions adopted to prepare $(Co_{50}Fe_{50})_{0.2}(TiO_2)_{99.8}$ catalyst powders. Specifically, 10 g of anatase TiO_2 was placed in the stainless-steel cylindrical vial of a SPEX Mixer/Mill 8000 together with two stainless-steel 12-g balls and the reactor sealed in Ar atmosphere. The reactor was clamped on the mechanical arm and swung at a milling frequency of about 14.6 Hz for 80 h. XRD analysis reveals that, as expected, prolonged BM induces the disappearance of the anatase TiO_2 phase and the formation of a mixture of brookite and rutile TiO_2 phases.

The obtained TiO_2 powder was placed inside a stainless-steel vial under Ar atmosphere together with a 12-g stainless-steel ball. The reactor was sealed using a cap equipped with gas-

tight pressure valves for the inlet and outlet of gases. Through several purge-refill cycles, the initial Ar atmosphere was replaced by a $CO:H_2$ gaseous mixture with composition 1:3. The final gas pressure was set at 0.3 MPa. Once clamped to the mechanical arm, the reactor was swung by the SPEX Mixer/Mill 8000.

No catalytic CO hydrogenation was observed.

SI.3. Characterization of milling dynamics

Experiments were performed using a suitably modified SPEX Mixer/Mill 8000 able to work at a frequency of vial motion variable between approximately 12.0 and 20 Hz. All experiments were carried out with a flat-ended stainless-steel vial having an internal volume of about 65 cm^3. The vial was loaded with a single stainless-steel 12-g ball and 8 g of TiO_2 powders previously milled for 80 h.

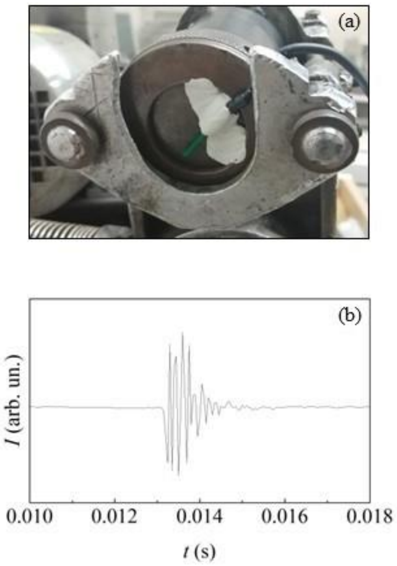

Fig. SI.3.1. (a) The piezoelectric sensor on the cap of the stainless-steel vial. (b) The signal generated by the piezoelectric transducer as a consequence of an impact between ball and vial. The signal intensity, I, is plotted as a function of time, t.

The milling dynamics was monitored using a common piezoelectric transducer suitably stuck to the stainless-steel vial bottom base or top cap. A picture is shown in Fig. SI.3.1a. The piezoelectric sensor gives rise to a rapidly decaying electric signal whenever an impact between ball and reactor occurs. A representative signal is reported in Fig. SI.3.1b for illustration purposes.

The sequence of piezoelectric signals provides information on both the impact frequency and velocity. The regularity of the sequence indicates that impacts are substantially inelastic. This is

due to the presence of a powder layer between the impacting surfaces that is compressed, which allows an almost complete dissipation of the ball mechanical energy.

The 8 g of catalyst powders used in CO hydrogenation experiments are enough to ensure inelastic impacts. Under these circumstances, the ball undergoes a regular and periodic motion between the opposite vial bases, with two collisions occurring per cycle. Indeed, after each impact, the ball remains in contact with reactor base, accelerates with the vial up to the highest vial velocity and, then, moves towards the opposite base as the vial slows down. Then, a new impact takes place.

The time period between two consecutive signals corresponds to the time interval between two impacts. Combined with the numerical simulation of the vial motion, it allows estimating the velocity with which the ball impacts on the reactor. In particular, the velocity of each impact was estimated from the time interval between the vial reaching its maximum oscillation amplitude and the impact between ball and vial. The latter time interval can be measured using a magnetic proximity sensor. The experimental methods are explained in greater detail in previous work.

SI.4. Powder trapped in individual impacts

A SPEX Mixer/Mill 8000 mill was used with a standard stainless-steel flat-ended vial loaded with a single 12-g stainless-steel ball and approximately 8 g of TiO_2 powder previously milled for 80 h. The mill was operated at an impact frequency of about 14.6 Hz, which results in an impact velocity of about 4.2 m s^{-1} [33,49]. Experiments were performed operating the mill only for approximately 10 s. Within this time interval, about 140 impacts take place at each end of the vial. After the very short milling period, the vial was opened and suitably examined together with the powder. It was possible to find 18 individual powder compacts formed by powder compression at impact, 8 stuck to the surface of the vial bases and 10 dispersed in the mass of powder. All of them can be expected to have undergone only a single impact. They were carefully collected and stereographically analyzed using an optical microscope.

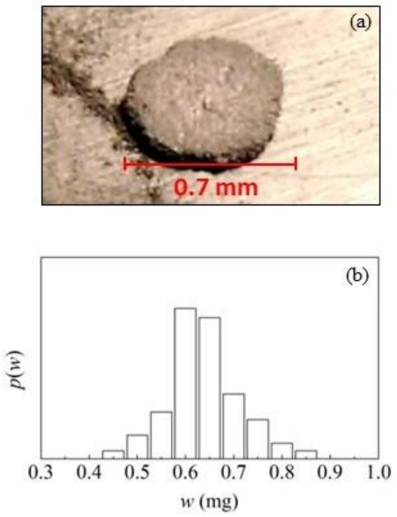

Fig. SI.4.1. (a) A typical powder compact formed by the dynamic compaction of loose catalyst powder induced by an impact between ball and vial. (b) Mass distribution of powder compacts.

A typical image is shown in Fig. SI.4.1a. The powder compacts were also weighed with a precision analytical balance. The results are shown in Fig. SI.4.1b. It can be seen that the mass distribution is quite narrow, with an average value of about 0.63 mg.

SI. 5. Catalytic activity

The CO hydrogenation reaction can be assumed to have a kinetics roughly described by the second-order rate law

$$r_{\mathrm{hydr}} = k\, p_{\mathrm{CO}}\, p_{\mathrm{H_2}}, \qquad \text{(SI.5.1)}$$

where r_{hydr} is the reaction rate, k is the apparent rate constant, and p_{CO} and $p_{\mathrm{H_2}}$ are the partial pressures of CO and H_2 gases respectively. The CO hydrogenation rate can be assumed to exhibit an Arrhenius-like dependence on temperature, T. Therefore, the apparent rate constant can be expressed as

$$k = A\, exp(-\, E_{\mathrm{a}}/R\, T), \qquad \text{(SI.5.2)}$$

where A is the frequency factor, E_{a} is the apparent activation energy and R is the universal gas constant.

Being the partial pressures p_{CO} and $p_{\mathrm{H_2}}$ the same, the reaction rate can increase as a consequence of a decrease of the activation energy E_{a} or an increase of temperature T.

In the former case, the ratio between the reaction rates can be expressed as

$$r_{\mathrm{hydr,2}}/r_{\mathrm{hydr,1}} = exp[-\,(E_{\mathrm{a,2}} - E_{\mathrm{a,1}})/R\, T]. \qquad \text{(SI.5.3)}$$

If the ratio $r_{\mathrm{hydr,2}}/r_{\mathrm{hydr,1}}$ is equal to 1×10^3 at 300 K, the difference of activation energies $E_{\mathrm{a,2}} - E_{\mathrm{a,1}}$ must be equal to about -17.2 kJ mol^{-1}. This means that, if the activation energy $E_{\mathrm{a,1}}$ of the CO hydrogenation process over catalyst powders under conventional conditions is equal to 150.0 kJ mol^{-1}, the activation energy $E_{\mathrm{a,2}}$ associated with catalyst powders that undergo impacts is equal to 132.8 kJ mol^{-1}.

Along the same line, if the ratio $r_{\mathrm{hydr,2}}/r_{\mathrm{hydr,1}}$ is equal to 1×10^6 at 300 K, the difference of activation energies $E_{\mathrm{a,2}} - E_{\mathrm{a,1}}$ must be equal to about -34.4 kJ mol^{-1}. This means that, if the activation energy $E_{\mathrm{a,1}}$ of the CO hydrogenation process over catalyst powders under conventional conditions is equal to 150.0 kJ mol^{-1}, the activation energy $E_{\mathrm{a,2}}$ associated with catalyst powders that undergo impacts is equal to 115.6 kJ mol^{-1}.

In the case the reaction rate difference depends on temperature, the ratio between the reaction rates can be expressed as

$$r_{\mathrm{hydr},2}/r_{\mathrm{hydr},1} = exp(-E_{\mathrm{a}}/R\,T_2 + E_{\mathrm{a}}/R\,T_1). \quad \text{(SI.5.4)}$$

As in the previous case, if the ratio $r_{\mathrm{hyd}\ ,2}/r_{\mathrm{hydr},1}$ is equal to 1×10^3, the activation energy E_{a} is equal to 150.0 kJ mol^{-1}, and the temperature T_1 at which the CO hydrogenation process over catalyst powders under conventional conditions is equal to 300 K, the temperature T_2 at which the CO hydrogenation reaction proceeds over catalyst powders that undergo impacts must be equal to about 340 K.

Along the same line, if the ratio $r_{\mathrm{hydr},2}/r_{\mathrm{hydr},1}$ is equal to 1×10^6, the activation energy E_{a} is equal to 150.0 kJ mol^{-1}, and the temperature T_1 at which the CO hydrogenation process over catalyst powders under conventional conditions is equal to 300 K, the temperature T_2 at which the CO hydrogenation reaction proceeds over catalyst powders that undergo impacts must be equal to about 390 K.
